# Supplementary material for: Disparities by race and insurance-status in declines in pediatric ED utilization during the COVID19 pandemic
Source: PLoS One. 2022 Feb 9;17(2):e0262490. doi: 10.1371/journal.pone.0262490 (PMC8827414; doi:10.1371/journal.pone.0262490)
Supplement: Attachment — Submitted filename: PLoS One Response to Reviewers.docx [file pone.0262490.s001.docx]

**RESPONSE TO EDITOR & REVIEWERS**

***We thank theeditor reviewers for their careful reading of our manuscript, and for excellent and thoughtful comments that have helped us strengthen the manuscript. We list below how we responded to each individual suggestion or concern raised by editor and reviewers.***

RESPONSE TO EDITOR

When submitting your revision, we need you to address these additional requirements.

1. Please ensure that your manuscript meets PLOS ONE's style requirements, including those for file naming. The PLOS ONE style templates can be found at 
<https://journals.plos.org/plosone/s/file?id=wjVg/PLOSOne_formatting_sample_main_body.pdf> and 
<https://journals.plos.org/plosone/s/file?id=ba62/PLOSOne_formatting_sample_title_authors_affiliations.pdf>.

***Thank you. We have tried to adhere to this format in our revised paper. We will be happy to make further changes if we missed any formatting instructions.***

2. Please update your submission to use the PLOS LaTeX template. The template and more information on our requirements for LaTeX submissions can be found at <http://journals.plos.org/plosone/s/latex>.

***We have used the Latex template (and have inserted the tables in the document as per instructions, but the figure is being submitted separately). We have now submitted a version with tracked changes in response to reviewer comments, and a clean version (as per instructions), and both versions follow the Latex template.***

3. Thank you for stating in your Funding Statement: 
 [The authors were partially supported through an internal grant by the School of Public Health, University of Alabama at Birmingham. The funders had no role in study design, data collection and analysis, decision to publish, or preparation of the manuscript.]
  
Please provide an amended statement that declares *all* the funding or sources of support (whether external or internal to your organization) received during this study, as detailed online in our guide for authors at <http://journals.plos.org/plosone/s/submit-now>.  Please also include the statement “There was no additional external funding received for this study.” in your updated Funding Statement.

Please respond by return e-mail so that we can amend your financial disclosure and competing interests on your behalf.

***We have now amended the funding information as follows (please note, as per the instructions here we also emailed this information to PLoS One separately in return email). The amended statement reads “This research was partially supported by an internal grant from the Office of the Dean, Lister Hill Center for Health Policy, and Sparkman Center for Global Health at the School of Public Health, UAB. No other external funding was received for this grant. The funders provided support in the form of salaries for the statistician for the project, Dr Nianlan Yang, and for graduate research assistants, but did not have any additional role in the study design, data collection and analysis, decision to publish, or preparation of the manuscript. While corresponding author Dr Bisakha Sen, and co-author Dr. Anne Brisendine are faculty in the Department of Health Care Organization & Policy,  School of Public Health, UAB, they have no direct affiliation with the Office of the Dean, Lister Hill Center for Health Policy, or Sparkman Center for Global Health, and this does not impact the authors’ adherence to  PLOS ONE policies on sharing data and materials.”***

4. Thank you for providing the following Funding Statement:  
[The authors were partially supported through an internal grant by the School of Public Health, University of Alabama at Birmingham. The funders had no role in study design, data collection and analysis, decision to publish, or preparation of the manuscript.].

We note that one or more of the authors is affiliated with the funding organization, indicating the funder may have had some role in the design, data collection, analysis or preparation of your manuscript for publication; in other words, the funder played an indirect role through the participation of the co-authors. 
If the funding organization did not play a role in the study design, data collection and analysis, decision to publish, or preparation of the manuscript and only provided financial support in the form of authors' salaries and/or research materials, please review your statements relating to the author contributions, and ensure you have specifically and accurately indicated the role(s) that these authors had in your study in the Author Contributions section of the online submission form. Please make any necessary amendments directly within this section of the online submission form.  Please also update your Funding Statement to include the following statement: “The funder provided support in the form of salaries for authors [insert relevant initials], but did not have any additional role in the study design, data collection and analysis, decision to publish, or preparation of the manuscript. The specific roles of these authors are articulated in the ‘author contributions’ section.”

If the funding organization did have an additional role, please state and explain that role within your Funding Statement.

Please also provide an updated Competing Interests Statement declaring this commercial affiliation along with any other relevant declarations relating to employment, consultancy, patents, products in development, or marketed products, etc.

Within your Competing Interests Statement, please confirm that this commercial affiliation does not alter your adherence to all PLOS ONE policies on sharing data and materials by including the following statement: "This does not alter our adherence to  PLOS ONE policies on sharing data and materials.” (as detailed online in our guide for authors <http://journals.plos.org/plosone/s/competing-interests>). If this adherence statement is not accurate and  there are restrictions on sharing of data and/or materials, please state these. Please note that we cannot proceed with consideration of your article until this information has been declared.

***Thank you. As you will see in our response to point 3, in our updated Funding statement we have provided these clarifications. We have also updated the Competing Interests statement.***

5. We note that you have stated that you will provide repository information for your data at acceptance. Should your manuscript be accepted for publication, we will hold it until you provide the relevant accession numbers or DOIs necessary to access your data. If you wish to make changes to your Data Availability statement, please describe these changes in your cover letter and we will update your Data Availability statement to reflect the information you provide.

***We are preparing to share the data needed to replicate our results via OpenICPSR (***[***https://www.openicpsr.org/***](https://www.openicpsr.org/)***). We will complete the data upload process and share the needed information if the manuscript is accepted for publication by PLoS One.***

6. Please amend your list of authors on the manuscript to ensure that each author is linked to an affiliation. Authors’ affiliations should reflect the institution where the work was done (if authors moved subsequently, you can also list the new affiliation stating “current affiliation:….” as necessary).

***We have now added all author affiliations following the guidelines in the Latex template.***

RESPONSE TO REVIEWERS

Reviewer #1: This study evaluates the trends in pediatric emergency department visits in 2019 and 2020 (during the COVID-19 pandemic). The authors evaluate these trends in subgroups based on race, ethnicity, and insurance status. In addition, the study addresses an important topic given the well-known racial disparities further revealed by COVID, especially in the southern United States. Some issues with the methods and results reporting need to be clarified/addressed. Please see below for more specific comments by section:

Introduction:
• More references are needed to support a few of the statements made in the introduction.
o The 4th sentence ending in “…higher rates of ED use compared to the rest of the nation pre-pandemic” needs more support.

Thank you for this suggestion. We have included two more citations to support this.

o Additionally, the penultimate sentence in that section needs more detail regarding vaccination rates and disparities specific to the region, perhaps as it compares to the Northeastern states mentioned earlier.

We have followed the reviewer’s suggestion and included two citations on vaccination rates. Further, the suggestion about providing more details about COVID-19 vaccination rates in the Deep South states versus Northeastern states had been implemented, P.1 para 2 now includes the sentence “***Further, given the low COVID19 vaccination rates in the Deep South states – for example, Alabama and Mississippi at the end of September had fully vaccinated 52.0% and 53.2% of their respective populations compared to Pennsylvania with 68.5%or New York state with 75.3% fully vaccinated – this implies these states may remain vulnerable to the disruptions caused by COVID19 longer than other parts of the country.”***

Methods:
• Study site: Please provide some more detail about the study site including annual patient volume, tertiary/teaching, etc. to help the audience draw conclusions regarding generalizability of the results.

***Thank you for this suggestion. These details have now been provided in the opening paragraph of the Methods section, which now reads: “An observational study was conducted using data from the Children’s of Alabama ED (CoA-ED) -- the largest pediatric ED in the state, located in Birmingham, Jefferson County. CoA serves as a teaching hospital for the University of Alabama at Birmingham (UAB). CoA-Ed is Alabama’s main tertiary care center for pediatric patients, the state’s only designated Level 1 pediatric trauma center, and saw approximately 72,000 pediatric patients per year over 2015-2019.”***

• Study dates: I suspect the study periods were chosen to compare pre- and peri-pandemic data in a full 12 month period, however, please clarify rationale for selecting these dates.

***Thank you for this comment. The reviewer is absolutely correct regarding our motivation for choosing the dates, and in fact we used the wording from this comment to succinctly describe our motivation: “In order to compare pre and peri COVID-19-pandemic ED visit data for a full 12 month period, we selected January 1^st^ – December 31^st^, 2019 and 2020 for the study period.”***

• While the variables provided are laid out clearly, some more detail is necessary to develop a better picture of the dataset. If available, some more demographic data would be helpful to compare the 2019 and 2020 groups, specifically age.

***We have now included both age and gender distribution in Table 1. Further, following Reviewer 2’s suggestions, we have included raw numbers in addition to the percentages.***

• Please clarify further the 5-point acuity scale – is this ESI?

***Thank you for pointing this out. Yes it is ESI. This has now been clarified in Paragraph 2 of Materials & Methods, along with a reference for readers who wish to learn more about ESI.***

• Smoothing: Please provide some references for rationale.

***Thank you for this suggestion. Our primary reason for doing this was that we wished to compare date specific changes in ED visit volume in 2020 compared to 2019, but the dates fall on different days of the week in the two years, and it is well-documented that ED visits can vary across days of the week – particularly weekdays versus weekends. A 7-day average smooths these fluctuations – hence, the date-specific comparisons are not subject to spurious fluctuations due to being on different days of the week. We have now elaborated on this in Para.3 of the Materials & Methods section, and also provided several citations to support evidence that ED visit volume can fluctuate across days of the week.***

Results:
• Throughout this section, please specify the years for each month mentioned for consistency and clarification.

***As per the reviewer’s suggestion, we have now consistently mentioned year along with month in the Results section.***

• Consider combining the third and fourth paragraphs of this section to better incorporate the numbers (paragraph 4) alongside the text (paragraph 3).

***We tried to re-write this part as per the reviewer’s suggestion, but because the third paragraph still refers to Figure 1 (which we have now clearly stated at the beginning of that paragraph), while the fourth paragraph refers to Table 2, we felt that our attempts at combining these paragraphs actually made it more confusing. Hence, we eventually decided to keep the two paragraphs separate.***

• Table 1 – Clearly presented overall. Please provide the raw numbers alongside the percentages.

***We have now followed the reviewer’s suggestions and included raw numbers in Table 1.***

• Figure 1 – Please include the years on each graph. Per the title of the figure, the figures are comparing 2020 and 2019, but this should be clarified with labels.

***We have now edited the labels and the title as per the reviewer’s suggestion.***

• Table 2 – Very important table! Please clarify dates in the first column (All 12 months, up to March 13, after March 13) with years and specific time periods (e.g. January 1, 2020-December 31, 2020; January 1, 2020 – March 13, 2020; March 14, 2020-December 31, 2020). Additionally, please report 95% confidence intervals in this table. Worth highlighting this in the text (including abstract).

***Thank you for this suggestion. We have provided this additional information, including 95% CI, as suggested by the reviewer. We agree this makes the table more informative for the reader. We have also included the 95% CI information in the abstract. Further, we have included the 95% CI in the Results as well. There was some disagreement among our team about whether this made the Results section too cumbersome, however we eventually decided that this was necessary to make the written part consistent with Table 2.***

Discussion:
• The discussion here is interesting and keeps with the paper’s focus. The authors have done a good job addressing all the major points. However, parts of the discussion would benefit from some more detail and support from the literature.

• The following statements should be discussed in more detail and/or supported with findings from the literature:
o The first sentence in the discussion – “Growing literature has documented declines…” – please cite the literature here.

***Thank you for catching this. We have now re-cited the literature after this statement, which should make it more convenient for readers.***

o Supplementary Figure 1 by Adjemian et al. was not included in the document.

***Thank you for pointing out this omission. We have now included the link to this Supplementary Figure (https://stacks. cdc.gov/view/cdc/104808) in the text itself (though we can move it to the Bibliography if editors so instruct).***

o Please expand on inability to document specific health conditions. As the authors mention, this is an important limitation.

***Thank you for this comment. We have now explained in the “limitations” paragraph that the specific challenge was that the “EMR data received from CoA-ED did not include ICD-10 codes for diagnosis or claims”. While there were notes on patient symptoms and conditions in the EMR data we received, these did not follow any structured format or wording. Thus, based on these data, it was not possible to reliably classify whether the patient was in the ED due to COVID19 infection.***

o The sentence – “…since Alabama shares several socio-demographic and economic characteristics with other Deep South states” – please support this statement as well.

***Thank you for this suggestion. We have now provided citations that support socio-demographic and economic similarities between the Deep South states both in the Introduction (Para 1) and after the particular sentence the reviewer mentioned in the Discussion.***

Additionally, there are some minor grammatical and typographic errors (i.e. U.S. instead of United States on first use in the introduction) – please check for these throughout the document.

***Thank you. We have made these changes (including using the expanded “United States” at first mention in abstract and the Introduction).***

Reviewer #2: The authors set out to explore the differences in the utilization of the ED at a major pediatric emergency department in Alabama during the Covid-19 pandemic based on racial and socioeconomic factors. The authors found a significantly larger decline in ED utilization by the African-American and publicly insured or self-insured patients in comparison to non-Hispanic white and privately insured patients, respectively. These findings inform the reader of the further worsening of healthcare disparities in the pandemic environment and suggest an exacerbation of unmet healthcare needs of vulnerable populations.

1. Parts of the manuscript are somewhat difficult to read. I recommend reviewing the revised manuscript for grammar and syntax errors.

***Thank you for pointing this out. We have now carefully reviewed the entire manuscript, and believe we have been able to correct all grammar and syntax errors.***

2. It appears that the authors chose to group publicly- and self-insured patients together. What was the breakdown of those groups? Are those populations truly comparable enough to be considered together?

***This is a very good point, and our team had actually debated this at the start of the analyses. We finally decided to group them together because in Alabama low-income children tend to “churn” between being publicly insured and self-insured/uninsured. The self-insured group was too small to warrant separate analyses but omitting them altogether felt like leaving out an important group of vulnerable children. We have now included raw numbers as well as percentages in Table 1, and it can be seen that there were 81320 publicly insured children and 4143 self-insured children in the PUBLIC-SELF group, making the total 85463, of which 4.8% were self-insured. However, to address the reviewer’s concern, we also conducted sensitivity analyses with just the publicly insured children (omitting self-insured) and PRIVATE (we state this at the end of Para.3 in the Methods section), and we found virtually identical results (we state this in the final section of Results). We do not show those results in the paper, but they are given below for the editors and reviewers to look at.***

**Results from Comparing PUBLIC-SELF with PRIVATE versus Just PUBIC with PRIVATE.**

|  | **Mean Percentage Change in total ED Visits Between 2019 and 2020** | | | | | |
| --- | --- | --- | --- | --- | --- | --- |
|  | **Publicly Insured or self-insured Children** | **Privately Insured Children** | **P-value from T-test** | **JUST Publicly Insured Children** | **Privately Insured Children** | **P-value from T-test** |
| **Jan 1^st^ -Dec 31^st^ 2020 compared**  **to 2019** | **-36.32** | **-27.63** | **P<0.001** | **-36.24** | **-27.63** | **P<0.001** |
|  |  |  |  |  |  |  |
| **Jan 1^st^- March 13^th^ 2020 compared**  **to 2019** | **-3.98** | **-3.90** | **P=0.904** | **-3.59** | **-3.90** | **P=0.851** |
|  |  |  |  |  |  |  |
| **March 13^th^-Dec 31^st^ 2020 compared**  **to 2019** | **-43.48** | **-32.87** | **P<0.001** | **-43.46** | **-32.87** | **P<0.001** |
|  | **Mean Percentage Change in High-severity ED Visits Between 2019 and 2020** | | | | | |
|  | **Publicly Insured or self-insure Children** | **Privately Insured Children** | **P-value from T-test** | **JUST Publicly Insured Children** | **Privately Insured Children** | **P-value from T-test** |
| **Jan 1^st^ -Dec 31^st^ 2020 compared**  **to 2019** | **-21.72** | **-20.01** | **P=0.04** | **-21.65** | **-20.01** | **P=0.04** |
|  |  |  |  |  |  |  |
| **Jan 1^st^- March 13^th^ 2020 compared**  **to 2019** | **0.18** | **-4.01** | **P=0.016** | **0.97** | **-4.01** | **P=0.004** |
|  |  |  |  |  |  |  |
| **March 13^th^-Dec 31^st^ 2020 compared**  **to 2019** | **-26.56** | **-23.54** | **P=0.001** | **-26.61** | **-23.54** | **P=0.006** |

3. The age range of the patients was not defined. Was there a difference among groups? This could be another variable relevant for analysis.

***We agree that age-range of patients is useful information. The age-range was 0-<19 years old, and that information has been added in Materials & Methods. Further, in Table 1, we have now included the breakdown of patients in age range 0-3, 3-6, 6-12 and 12-18. However, we decided that adding a third set of graphs by age-range would make the paper too cumbersome, and that more in-depth analyses of changes by age was best left for future work.***

4. Was there an overlap in the two sets of groups (AA and NHW and between PUBLIC-SELF and
PRIVATE)? In other words, for instance, was there a difference in the AA + PUBLIC-SELF and AA + PRIVATE? Is possible that the difference is more related to the race or the insurance status?

***This is a valid concern that may occur to many readers. However, in case of Alabama, a substantial portion of Medicaid and Children’s health Insurance Program enrollees are actually NHW. For example, here is a breakdown of race/ethnicity for all Alabama Medicaid enrollees (children, pregnant women and other adults) in 2018 out of a recent presentation made by Alabama Medicaid’s chief epidemiologist.***

Medicaid Eligibles By Race

|  | Race |
| --- | --- |
| Black | 429798 |
| White | 423007 |
| Other | 120050 |
| Hispanic | 51155 |

***We have also spelled out the breakdown of NHW in our Results section, and have also included the sentence “In further breakdowns not shown in the table, it was seen that among children in PUBLIC-SELF, 37.5% were NHW (36.4% of patients on Medicaid, 54.6% of patients on CHIP, and 34.6% of self-pay patients), while among those in PRIVATE, 74.9% were NHW.” While the majority of Medicaid and This should alleviate concerns about too much overlap between AA and PUBLIC-SELF that is driving the results.***

5. Results Section, paragraph 2, last sentence: the statement is somewhat ambiguous, as it appears to imply that the groups were combined (AA and PUBLIC vs. NHW and PRIVATE).

***Thank you for this suggestion. We have reworded that sentence to make it clear that we are still comparing NHW with AA, and PUBLIC-SELF with PRIVATE.***

6. Page 7, Discussion, sentence starting with: While this paper’s findings of steep declines… is unclear the way it is written.

***Thank you for catching this grammatical error! We have now corrected and re-written it, so that it should be clear.***

7. The authors make a great point discussing the lower rates of utilization of telemedicine among African American and low income children. However, is there any data on urgent care use? Is it possible that the people were more likely to take their children to urgent care facilities due to shorter lines and more predictable wait times, especially for low-severity visits?

***This is an excellent point. We searched for articles on pediatric urgent care use during COVID19, but were unable to find any articles. And of course, the ED EMR data does not permit deciphering whether children went to urgent care instead. Hence in our paragraph on Limitations we have now explicitly said that we cannot decipher “whether care was being accessed in alternate settings such as urgent care or office visits.”***
