# Supplementary material for: Disparities by race and insurance-status in declines in pediatric ED utilization during the COVID19 pandemic
Source: PLoS One. 2022 Feb 9;17(2):e0262490. doi: 10.1371/journal.pone.0262490 (PMC8827414; doi:10.1371/journal.pone.0262490)
Supplement: Attachment — Submitted filename: PLoS One Response to Reviewers ROUND2.docx [file pone.0262490.s002.docx]

PONE-D-21-26799R1

Disparities by Race and Insurance-Status in Declines in Pediatric ED Utilization During the COVID-19 Pandemic.

PLOS ONE

**Thank you for this opportunity to revise and resubmit our manuscript. We believe we have been able to address the remaining concerns, and the manuscript is further improved. We describe below how we addressed the concerns that remained.**

In the next revision, please:
1) edit text to clarify the comparisons between AA and NHW, and between Public-self and Private throughout to avoid any confusions as suggested by Reviewer 2. For example, the last sentence in the 2nd paragraph of the Results section, "Percentage declines were consistently larger for AA and PUBLIC-SELF than NHW and PRIVATE." This is confusing and you did not revise as suggested by Reviewer 2. This is one example but please read the entire manuscript to improve clarify of writing.

**Thank you for catching this omission. We have revised that sentence to read *“Percentage declines were consistently larger for AA compared to NHW, and for PUBLIC-SELF compared to PRIVATE.”* We also read the rest of the document carefully and made some more clarifying changes. For example, we edited the Conclusion paragraph for to improve clarity.**

2) The author's response to Reviewer 2's question regarding the overlap between AA+Public-self and AA+Private is not convincing. Please provide results for the direct comparison between these 2 subgroups in table and in text.

**We apologize for not fully understanding the reviewer concern the first time. We have now added the additional sub-analyses the reviewer was looking for, by comparing the PUBLIC-SELF and PRIVATE subgroups within the racial categories of AA and NHW. Specifically, we repeated the t-tests for mean differences in change in visits patients who were African-American and privately insured (AA_PRIVATE) versus who were African-American and public or self-insured (AA_PUBLIC-SELF), and mean differences for Non-Hispanic White patients who were privately insured (NHW_PRIVATE) and**

**Non-Hispanic White patients who were publicly insured or self-insured patients (NHW_PUBLIC-SELF). We were able to confirm that there were statistically significant, larger differences in mean declines for public and self-insured visits compared to private visits within each race category. We believe adding this analysis definitely improves the paper, and we are grateful for the suggestion!**

**We have described this additional analysis in the Materials & Methods section (p. 4 & p.5). We have added a new table (Table 3) to show those results, and also added a new paragraph with the Results. We include that paragraph and the table here as well:**

**“*T-test results comparing mean date-specific percentage changes in total visits for AA_PRIVATE versus AA_PUBLIC-SELF, and for NHW_PRIVATE versus NHW_PUBLIC-SELF are shown in Table 3. These results indicated that mean date-specific percentage declines in 2020 compared to 2019 were significantly larger for AA_PUBLIC-SELF than AA_PRIVATE (-40.07% [95% CI: -42.3, -37.9] compared to -34.15% [95% CI: -36.5, -31.8], p<0.001, and were also significantly larger for NHW_PUBLIC-SELF than NHW_PRIVATE (-31.95% [95% CI: -33.9, -29.9] compared to -25.95% [95% CI: -27.6, -24.3], p<0.001). As with previous results, the declines were larger and the differences in declines were more significant after March 13, 2020.”***

**Table 3: Differences in the Mean Date-Specific Percentage Changes in 2020 compared to 2019 for Total ED Visit Counts between Privately Insured & Publicly/Self-Insured Children, by Race.**

|  | **Mean Percentage Change in Total ED Visits Between 2019 and 2020**  **[95% C.I.]** | | | | | |
| --- | --- | --- | --- | --- | --- | --- |
|  | **African-American Children** | | | **Non-Hispanic White Children** | | |
|  | **Publicly Insured or Self-Insured Children** | **Privately Insured Children** | **P-value from T-test** | **Publicly Insured or Self-Insured Children** | **Privately Insured Children** | **P-value from T-test** |
| **Jan 1-Dec 31, 2020 compared**  **to 2019** | **-40.07**  **[-42.3, -37.9]** | **-34.15**  **[-36.5, -31.8]** | **P<0.001** | **-31.95**  **[-33.9, -29.9]** | **-25.95**  **[-27.6, -24.3]** | **P<0.001** |
|  |  |  |  |  |  |  |
| **Jan 1-Mar 13, 2020 compared**  **to 2019** | **-6.82**  **[-10.6, -3.0]** | **-1.82**  **[-6.7, 3.1]** | **P=0.03** | **-1.87**  **[-4.7, 1.0]** | **-4.10**  **[-5.9, -2.3]** | **P=0.28** |
|  |  |  |  |  |  |  |
| **Mar 13-Dec 31, 2020 compared**  **to 2019** | **-47.10**  **[-48.7, -45.5]** | **-40.99**  **[-42.8, -39.2]** | **P<0.001** | **-38.31**  **[-39.8, -36.8]** | **-30.57**  **[-31.9, -29.2]** | **P<0.001** |

3) Please giving the manuscript another read through for grammar and syntax prior to final submission as suggested by Reviewer 1.

**Thank you. We have done a thorough read for spelling, grammar, and syntax as suggested.**
